# Intravenous Magnesium Sulfate for Pain Management in Patients with Acute Renal Colic; a Randomized Clinical Trial

**Published:** 2020-01-28

**Authors:** Alireza Majidi, Fatemeh Derakhshani

**Affiliations:** 1 Emergency Department, Clinical Research Development Unit, Shohadaye Tajrish Hospital, Shahid Beheshti University of Medical Sciences, Tehran, Iran

**Keywords:** Magnesium sulfate, morphine, renal colic, pain management, emergency service, hospital

## Abstract

**Introduction:**

It seems that magnesium (Mg) sulfate can be helpful in controlling the acute pain caused by the stone passing from the ureter by reducing ureter smooth muscle contractions. The present study has been designed with the aim of assessing the role of Mg sulfate in controlling the renal colic pain in emergency department (ED).

**Methods:**

This double blind randomized clinical trial was performed on 18 to 60 year-old patients with acute renal colic presenting to the ED of a teaching hospital during 1 year. Patients were allocated to one of the 2 groups receiving either IV morphine or IV Mg sulfate using block randomization method and were then compared regarding pain control characteristics and probable side effects.

**Results:**

90 patients with the mean age of 37.34 ± 12.10 (18 – 60) years were divided into 2 equal groups. The 2 groups were in a similar condition regarding mean age (p = 0.168), sex distribution (p = 0.267), underlying disease (p = 0.414) and alcohol and drug abuse (p = 0.524). Mean pain scores of the patients based on VAS were not significantly different between the 2 groups on admission and 20, 30, 60, 120, and 180 minutes after drug administration. Success rate in reducing the pain by at least 3 points on VAS was equal and 91.1% for both groups on the 20^th^ minute and reached 100% on the 30^th^ minute for both groups. The number of cases that were pain-free on the 20^th^ minute was significantly higher in the morphine groups (31 versus 16 patients, p = 0.004). However, on the 30^th^ minute both groups experienced a similar condition in this regard (39 versus 29, p = 0.063). None of the patients in the 2 groups experienced the studied side effects.

**Conclusion:**

It seems that Mg sulfate, as a muscle relaxant agent, can be considered as a safe adjunct medication in controlling the pain of renal colic patients in the ED.

## Introduction

Pain is one of the most common causes of patients visiting emergency departments (EDs) and pain control is one of the important responsibilities of the specialists in this department (1). In the time interval from 2006 to 2009, there was an increase in the prevalence of renal colic from 289 to 305 cases for each 100000 population in the United States, part of which might be due to increased use of more accurate diagnostic tools such as computed tomography (CT) scan (2). 

Choosing an efficient drug with easy administration, few side effects, and rapid acting in pain management of these patients, is of great interest.

Main drugs used in managing renal colic pain are categorized into 2 major groups of non-steroid anti-inflammatory drugs (NSAIDs) and opioids. It seems that tocolytic drugs such as magnesium (Mg) sulfate can be helpful in decreasing the pain caused by the stone passing from the ureter by reducing ureter smooth muscle contractions. By preventing calcium from entering through the cell membrane of smooth muscles, Mg sulfate can reduce calcium, which is required for muscle contraction (3, 4). In addition, by reducing acetyl choline in neural terminals Mg sulfate can decrease muscle contractions (5). The role of Mg sulfate in decreasing pains due to surgeries has been confirmed in various studies (6-8).

The results of the study by Delavar Kasmaei et al. regarding comparison of Mg sulfate and ketorolac in controlling migraine headaches were indicative of similar effects of both drugs in the acute phase and better performance of Mg sulfate 1 hour and 2 hours after drug administration (9). Additionally, Jokar et al. have emphasized the role of Mg sulfate as an adjunct medication in controlling renal colic and reducing the need for opioid drugs (10). Mg sulfate has been found to be safe and effective in controlling neuropathic pains due to cancer (11). However, Kothari et al. have estimated the power of Mg sulfate to be lower than short-acting opioids (12). Studies regarding effectiveness and safety of Mg sulfate‌ in controlling patients’ pain are still ongoing, especially in the ED. Therefore, the present study has been designed and performed with the aim of assessing the role of Mg sulfate in controlling the pain caused by renal colic in ED.

## Methods:

***Study design and setting***

The present double blind randomized clinical trial was performed on patients with acute renal colic presenting to the ED of Shohadaye Tajrish Hospital, Tehran, Iran, from March 2017 to March 2018. Protocol of the study was approved by the ethics committee of Shahid Beheshti University of Medical Sciences under the number SBMU.MSP.REC.1396.2548 and was registered on the Iranian clinical trial registry under the number IRCT20171206037774N1. The researchers adhered to research ethics and confidentiality of patient data based on the recommendations of Helsinki Declaration. Informed consent was obtained from the patients for participating in the study.

Participants

Patients with acute renal colic in the age range of 18 – 60 years were studied without any sex limitation. Patients with known arrhythmia or heart block; cardiac failure, myocardial injuries; kidney failure; hepatitis; cardiac glycoside recipients; those consuming alcohol, opioid drugs, anti-anxiety medications, barbitorates, anti-depressants, anti-psychotic and sleep medications, and calcium channel blockers; those affected with myasthenia gravis and other neuromuscular diseases; pregnant women or those suspected with pregnancy; those having underlying bradycardia (heart rate less than 60 /minute) and long QRS interval; patients who did not sign the consent form; and cases with sensitivity to morphine or Mg sulfate were excluded from the study. In addition, those who had received any kind of analgesics or sedatives during the 6 hours prior to presentation to the ED were also excluded.

Intervention

After history taking and clinical examination, pain score of the patients was evaluated based on visual analogue scale (VAS) and morphine sulfate with 0.1 mg/kg dose was intravenously injected for all the patients via the median cubital vein during 1 minute. If the pain persisted 10 minutes after receiving morphine with a pain score over 6 and considering the inclusion and exclusion criteria, patients were allocated to either intravenous (IV) Mg sulfate (2cc of 50% solution that was diluted with normal saline solution until reaching 100 ml injected during 15 minutes) or IV morphine (0.1 mg/kg dose) groups via block randomization using random numbers table. Double dummy method was used for blinding, which means that recipients of injected morphine simultaneously received 100 ml normal saline and those receiving Mg also received IV distilled water with the same volume and speed as morphine.

The whole mentioned process was done under constant cardiac, respiratory and blood pressure monitoring and pulse oximetry under the supervision of the senior emergency medicine resident who was in charge of performing the study but was blind to the type of drug administered.

Considering the random method, injectable drugs were prepared in advance in syringes with the same color and with the same volume by a nurse who helped in performing the study and was blind to the pain severity and other characteristics of the patients on admission. Pain severity of the patients was reevaluated and recorded 20, 30, 60, 120, and 180 minutes after drug administration. If the patients’ pain was not controlled after being allocated to a study group and receiving the allocated medication, patients received reminder doses of morphine and were excluded from the study, but they were included in the statistical analyses in order to adhere to the intention to treat method.

Data gathering

Baseline characteristics of the patients, pain severity based on VAS on admission and 20, 30, 60, 120, and 180 minutes after drug administration and probable side effects such as nausea, vomiting, vertigo, shortness of breath, allergy, and respiratory depression were gathered and recorded for all patients by the senior emergency medicine resident in charge of the patient and under supervision of the attending physician on duty using a pre-designed checklist.

In this study, 3 or more points drop in the pain score based on VAS was considered as treatment success. Suspicion to renal colic was made by the senior resident in charge of the patient based on clinical symptoms including acute and sudden pain with a colic nature and its radiation to the groin or the lower part of the abdomen and also the testes with or without nausea, vomiting, sweating, paleness, burning urine, frequent urination, urinary urgency, and blood in urine, and was confirmed using ultrasonography or abdominal and pelvic computed tomography (CT) scan as well as urinalysis at the first possible opportunity that the patient could bear it.

Pain score was assessed using VAS. This scale consists of a 10-cm ruler on which 0 indicates absence of pain and 10 indicates the most severe pain possible.

***Outcomes***

The studied outcomes included success in pain management and probable side effects due to prescription of drugs.

Statistical analysis 

For statistical analysis, SPSS software version 20 was used and for comparing the results between the 2 groups, t-test, Mann-Whitney, Chi square, or Fisher’s exact tests were applied. The findings were reported as mean and standard deviation (SD) or frequency and percentage. P value less than 0.05 was considered as level of significance. The person performing statistical analyses was also blind to the drugs received by the groups.

## Results:

*Baseline characteristics of the studied patients*

**Figure 1:**
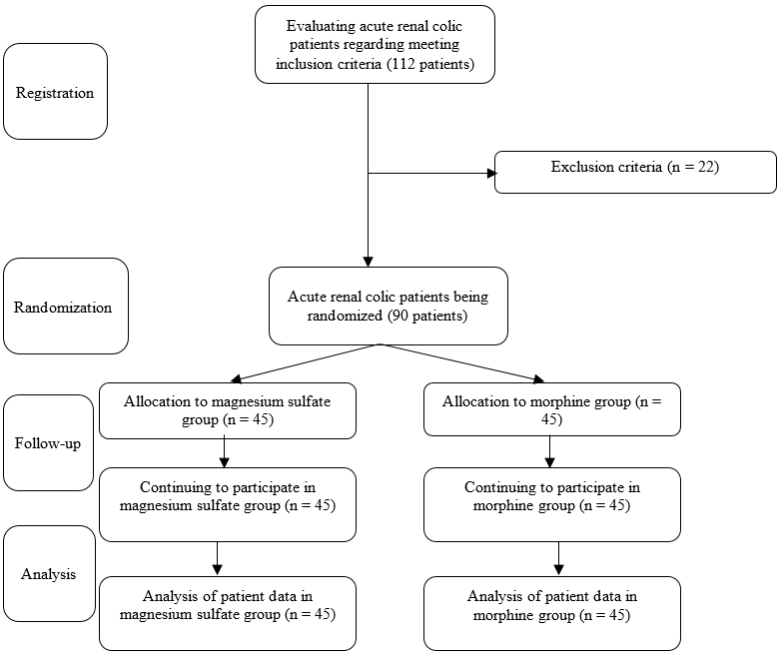
Flowchart of the study

Finally, 90 patients with the mean age of 37.34 ± 12.10 (18 – 60) years were randomly allocated to one of the 2 groups receiving either Mg sulfate (45 patients) or morphine sulfate (45 patients) (figure 1). Table 1 has compared the clinical data of the studied groups. The 2 groups were in a similar condition regarding mean age (p = 0.168), sex distribution (p = 0.267), being affected with underlying illnesses (p = 0.414) and alcohol and drug abuse (p = 0.524).

***Outcomes***

**Figure 2:**
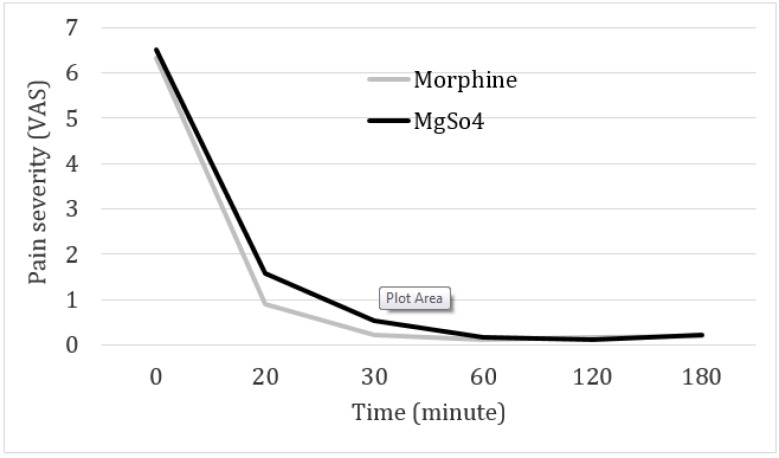
Pain reduction trend in the groups receiving morphine and magnesium at different studied times

Table and figure 2 have compared pain severity between the 2 groups on admission to ED and at other times. Mean pain score of the patients based on VAS on admission and 20, 30, 60, 120, and 180 minutes after drug administration were not significantly different between the 2 groups. Success rate in reducing the pain by at least 3 points on VAS was equal for both groups on the 20^th^ minute and was 91.1% (41 out of 45 patients in each group) and reached 100% on the 30^th^ minute for both groups. The number of cases that were pain-free (pain severity of 0) on the 20^th^ minute was significantly higher in the morphine groups (31 versus 16 patients, p = 0.004). Of course, on the 30^th^ minute both groups experienced a similar condition in this regard (39 versus 29, p = 0.063).

None of the patients in either group experienced any side effects including hypertension, allergy, drop in respiratory rate or reduced arterial blood oxygen saturation, and respiratory depression.

## Discussion:

Based on the findings of the present study, Mg sulfate showed a similar effect and an equal success rate in relieving the acute pain caused by renal colic in the patients presenting to ED when compared to morphine sulfate. However, it seems that the time interval to reach a pain-free status is a little longer for the group receiving Mg sulfate. None of the patients in either group experienced side effects in the prescribed doses in this study.

**Table 1:** Comparing the baseline characteristics of the studied patients in the groups receiving morphine and magnesium (Mg) sulfate

**Variables**	**Morphine (n = 45)**	**Mg sulfate (n = 45)**	**P value**
**Age (year)**			
Mean ± SD	39.1 ± 13.2	35.6 ± 10.8	0.168
**Sex **			
Male	27 (60.0)	32 (71.1)	0.267
Female	18 (40.0)	13 (28.9)	
**Drug abuse**			
Yes	27 (60.0)	17 (37.8)	0.414
No	18 (40.0)	28 (62.2)	
**Underlying illness**			
Yes	11 (24.4)	10 (22.2)	0.524
No	34 (75.5)	35 (77.7)	
**Vital signs (on admission)**			
Systolic BP (mmHg)	124.5 ± 12.3	122.3 ± 9.1	0.327
Diastolic BP (mmHg)	82.3 ± 11.4	80.0 ± 6.5	0.254
Heart rate (/minute)	75.1 ± 6.7	76.3 ± 4.0	0.320
Respiratory rate (/minute)	12.4 ± 0.9	12.7 ± 1.6	0.130
O_2_ Saturation (%)	94.3 ± 2.0	93.0 ± 12.0	0.457

Data are presented as mean ± standard deviation (SD) or frequency (%). BP: blood pressure.

**Table 2:** Comparing the pain score of the patients in the groups receiving morphine and magnesium (Mg) sulfate at different studied times

**Time **	**Morphine (n = 45)**	**Mg sulfate (n = 45)**	**P value**
**On admission**	6.33±0.71	6.51±0.76	0.253
**20** ^th^ ** minute**	0.90±2.0	1.58±1.9	0.102
**30** ^th^ ** minute**	0.24±0.68	0.53±0.84	0.077
**60** ^th^ ** minute**	0.11±0.32	0.18±0.44	0.413
**120** ^th^ ** minute**	0.18±0.39	0.13±0.34	0.566
**180** ^th^ ** minute**	0.20±0.40	0.22±0.42	0.799

Data are presented as mean ± standard deviation (SD).

Findings of the present study were similar to the results of Jokar et al. study in 2016, which evaluated and compared Mg sulfate with ketorolac in management of pain caused by renal colic and expressed that Mg sulfate can reduce the pain of patients with renal colic and decrease the need for additional morphine (10). Kocman et al. found that injection of a low dose of Mg sulfate significantly reduced the pain following surgery in patients that underwent laparoscopic cholecystectomy (13).

Mg sulfate has been identified as a safe and efficient drug in dilation of the bronchi in patients with severe asthma (14). Mg sulfate leads to loosening of the smooth muscles in the bronchial walls and therefore, dilation of their airways, a mechanism that may also be true regarding the smooth muscles of the ureter. In a study, Rezae et al. evaluated the effect of Mg sulfate injection on pain relief following cesarean section and stated that injection of 50 mg/kg Mg sulfate leads to pain reduction and lowers the need for other analgesic drugs (15).

On the other hand, a study carried out on women giving birth via cesarean section showed that intraspinal injection of 5% bupivacaine and Mg sulfate did not have much effect on elongation of analgesia. However, IV injection of Mg sulfate along with intraspinal injection of 5% bupivacaine showed better results (16).

In the present study, adding Mg sulfate to the standard treatment of patients with acute renal colic led to a decrease in pain severity and reduced need for additional morphine. In the future, Mg sulfate might be considered for establishing pain control protocols and sedation induction in patients with acute renal colic in the ED. However, we should not forget that prescription of Mg sulfate is very dose-dependent; therefore, choosing the proper dose is very important for maximum efficiency and minimum toxicity.

On the other hand, Mg sulfate should not be used in patients with known cardiac block, myocardial injury, severe kidney failure, hepatitis, and Addison’s disease and it is not recommended for use in patients with decreased kidney function, those who receive cardiac glycosides, patients with myasthenia gravis and other neuromuscular diseases, and when giving birth.

Yet, in cases that there is an acute pain not responding to available medications or they are banned for use or are not available in ED, we might be able to consider Mg sulfate as a proper alternative with few side effects.

Considering the obtained results and not seeing any significant side effect when using Mg sulfate drug as well as its easy administration, and on the other hand the problems caused by prescribing opioids, Mg sulfate can be used for adjunct treatment of patients with acute renal colic in ED to decrease patients’ pain severity. In addition, through simultaneous use of Mg sulfate, the rate of administration of other opioid drugs and their possible side effects can also be reduced.

## Limitations

Not homogenizing patients regarding size and location of stone could be counted as a limitation of the present study. In addition, by increasing sample size, various subgroup analyses based on sex or drug and alcohol abuse and etc. can be performed.

## Conclusion:

Based on the findings of the present study, Mg sulfate showed a similar effect and an equal success rate in relieving the acute pain caused by renal colic in the patients presenting to ED when compared to morphine sulfate. However, it seems that the time interval to reach a pain-free status is a little longer for the group receiving Mg sulfate.
